# Flexible Sensor Foil Based on Polymer Optical Waveguide for Haptic Assessment

**DOI:** 10.3390/s25226915

**Published:** 2025-11-12

**Authors:** Zhenyu Zhang, Abu Bakar Dawood, Georgios Violakis, Ahmad Abdalwareth, Günter Flachenecker, Panagiotis Polygerinos, Kaspar Althoefer, Martin Angelmahr, Wolfgang Schade

**Affiliations:** 1 Department of Fiber Optical Sensor Systems, Fraunhofer Heinrich Hertz Institute, Am Stollen 19H, 38640 Goslar, Germany; ahmad.abdalwareth@hhi.fraunhofer.de (A.A.); guenter.flachenecker@hhi.fraunhofer.de (G.F.); martin.angelmahr@hhi.fraunhofer.de (M.A.); wolfgang.schade@hhi.fraunhofer.de (W.S.); 2 Institute of Energy Research and Physical Technologies, Clausthal University of Technology, Am Stollen 19A, 38640 Goslar, Germany; 3 School of Engineering and Materials Science, Queen Mary University of London, London E1 4NS, UK; a.dawood@qmul.ac.uk (A.B.D.); k.althoefer@qmul.ac.uk (K.A.); 4 Department of Electrical and Computer Engineering, School of Engineering, Hellenic Mediterranean University, 71004 Heraklion, Greece; violakisg@hmu.gr; 5 Control Systems and Robotics Laboratory, Department of Mechanical Engineering, School of Engineering, Hellenic Mediterranean University, 71004 Heraklion, Greece; polygerinos@hmu.gr

**Keywords:** optical sensor, polymer waveguide, flexible sensor, tactile sensor, stiffness mapping, minimally invasive surgery

## Abstract

Minimally Invasive Surgery is often limited by the lack of tactile feedback. Indeed, surgeons have traditionally relied heavily on tactile feedback to estimate tissue stiffness - a critical factor in both diagnostics and treatment. With this in mind we present in this paper a flexible sensor foil, based on polymer optical waveguide. This sensor has been applied for real-time contact force measurement, material stiffness differentiation and surface texture reconstruction. Interrogated by a commercially available optoelectronic device, the sensor foil offers precise and reproducible feedback of contact forces up to 5 N, with a minimal detectable limit of 0.1 N. It also demonstrates distinct optical attenuation responses when indenting silicone samples of varying stiffnesses under controlled displacement. When integrated onto a 3D-printed module resembling an endoscopic camera and manipulated by a robotic arm, the sensor successfully generated spatial stiffness mapsof a phantom. Moreover, by sliding over structures with varying surface textures, the sensor foil was able to reconstruct surface profiles based on the light attenuation responses. The results demonstrate that the presented sensor foil possesses great potential for surgical applications by providing additional haptic information to surgeons.

## 1. Introduction

Developments in minimally invasive surgery (MIS) techniques have altered medical procedures by reducing the need for large incisions, thereby minimizing postoperative pain, accelerating recovery times, and reducing trauma for the patients. While these advancements have significantly improved patient’s experience during the surgical procedure, it brings with it new challenges for surgeons, most notably in relation to the lack of direct tactile feedback that are typically able to acquire through conventional surgical instruments such as grasping forceps and endoscopic cameras. This absence of real-time tactile feedback can hinder the surgeon’s ability to accurately evaluate tissue properties such as stiffness and texture, potentially putting patients at increased risk [1]. This highlights the need for sensor systems that can enhance the haptic response capabilities during MIS operations [2,3].

Addressing this need, research has increasingly been focused on the on the development of sensors that enable real-time tactile feedback and enhance human–machine interactions within surgical environments. Various sensing mechanisms, including piezoelectric [4], electrical resistance [5], capacitance [6], and camera-based [7] principles, have been extensively investigated in relation to their potential application for tactile sensing. Among these, optical sensors have emerged as particularly promising for surgical applications due to their miniaturized and compact size, low operational power consumption, and immunity to electromagnetic interference [8]. Indeed, optical fiber-based tactile sensors have been integrated into robotic grippers to improve human–machine interface functionality [9,10]. Other recent advancements include photonic tactile sensors based on fiber Bragg gratings [11,12,13], micromachined structures [14], and coated polymer optical fibers (POFs) [15]. Unlike photonic sensors that rely on spectral analysis, POF-based tactile sensors typically operate by evaluating the light signal intensity: tactile actuation alters the shape of the optical waveguide, leading to changes in transmitted light intensity, thus enabling force contact detection with a simplified, cost-effective measurement setup. These intensity-based sensors offer measurement precision comparable to photonic sensors that require laser-induced modulations within the waveguide along with expensive systems for spectral analysis [16,17].

However, intensity-based optical tactile sensors also have limitations: conventional POFs have rigid cylindrical geometries that restrict their mechanical deformation under external force contact, resulting in reduced sensitivity and insufficient response for tactile applications. To overcome this challenge, strategies such as integration of mechanical actuators [18] or functional coatings [15] have been proposed to amplify the tactile response. Mechanical actuators, however, tend to increase the overall sensor size, limiting their integration into compact MIS instruments, while functional coatings often consist of metallic materials, which can influence biocompatibility and potentially hinder deployment in biomedical applications.

Planar polymer optical waveguides (PPWs) offer a promising alternative. Most importantly, their planar geometry allows for a larger contact area compared to cylindrical POFs [19]. Also, when fabricated on flexible substrates, they can be integrated onto non-planar surfaces. For example, Yun et al. developed a flexible sensor array based on polymer waveguides that was capable of dynamic force sensing at multiple points and deployable as a wearable sensor device on human arms [20]. Nevertheless, their potential could be further explored by applying the PPWs on a flexible substrate on the lateral surface of existing MIS instruments. Ultimately, their capability to provide real-time feedback on tissue stiffness or surface texture remains to be explored.

In this paper, we present a flexible sensor foil based on a PPW. It enables direct quantitative evaluation of various tactile and haptic stimuli, without any requirement of additional functional coating layers or actuators. With its compact structure, it offers real-time feedback—a notable advantage in MIS scenarios in which quantitative haptic perception is an important key for improving surgical precision. Our contributions are threefold:

1. The design and fabrication of a miniaturized flexible polymer optical waveguide, compatible for integration with MIS tools.
2. Characterization of the sensor foil’s performance in response to contact forces and defined stiffness levels.
3. Demonstration of the sensor foil’s capability for stiffness mapping and surface texture profile reconstruction when integrated on a robot arm.

## 2. Sensing Principle

**Figure 1:**
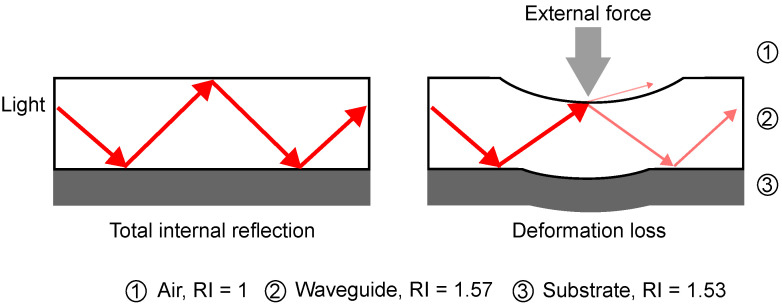
Light propagating behaviors (indicated by red arrows) and actuation due to bending loss. (**left**): Schematic illustration of total internal reflection within a multimode waveguide. (**right**): Illustration of bending-induced optical loss in a multimode waveguide. External force causes deformation of the flexible substrate, reducing the local curvature radius and increasing light leakage due to the violation of total internal reflection conditions.

Light propagation within a multimode waveguide is determined primarily by total internal reflection (TIR). In this context, light is confined within the higher refractive index region of the waveguide, ensuring efficient transmission with minimal optical loss [21]. This principle is illustrated on the left of Figure 1.

When an external force is applied, the multimode waveguide and the flexible substrate undergo mechanical deformation, leading to stretching and bending of the waveguide core structure. This deformation results in two principal effects: Firstly, the stretching of the waveguide results in an increase in the optical path length for higher-order modes, which typically located at the outer bending regions of the core. This stretching leads to a velocity increase in the light that is located at the outer modal rings of the core and a corresponding decrease in the propagation constant [22,23]. Secondly, the bending of the waveguide alters the incident angle of the guided light at the core–cladding interface. As this angle approaches or exceeds the critical angle for TIR, light is coupled into the cladding, producing attenuations. The right side of Figure 1 illustrates this process, in which an increase in external force causes increased deformation, thereby also increasing the optical attenuation due to bending. Consequently, the magnitude of the applied force can be quantitatively evaluated by monitoring the reduction in light intensity transmitted through the waveguide.

The numerical calculation of the bending loss $\alpha_{B}$ inside a multimode step-index cylindrical optical waveguide is described by the work of Gloge [23]:

(1) $$\alpha_{B}=2n_{core}k\left(\theta_{c}^{2}-\theta_{b}^{2}\right)exp\left[-\frac{2}{3}n_{core}kR{\left(\theta_{c}^{2}-\theta_{b}^{2}-\frac{2a}{R}\right)}^{\frac{3}{2}}\right]$$

Here, *R* is the bending radius, $n_{core}$ is the refractive index of the waveguide core, *k* is the free space wavenumber, $\theta_{c}$ and $\theta_{b}$ are the critical angles of TIR and the angle caused by the bending, respectively. Based on this equation, the bending loss increases with waveguide core radius *a*.

In response to this relationship, larger waveguide dimensions are selected to enable better optical transmission and to enhance sensitivity to bending-induced losses. With consideration of the material properties of photoresists, EpoClad50 (Microresist Technology GmbH, Berlin, Germany) with the maximum achievable layer thickness (50 μm) was therefore selected for fabricating the bending-sensitive PPW. The waveguide width was set at 500 μm to match the diameter of the POF (ESKA Fiber Strands, Edmund Optics, Ltd., Mainz, Germany) used for coupling of light into the waveguide core, thereby optimizing optical transmission and sensor performance for multimode bending evaluation.

## 3. Materials and Methods

**Figure 2:**
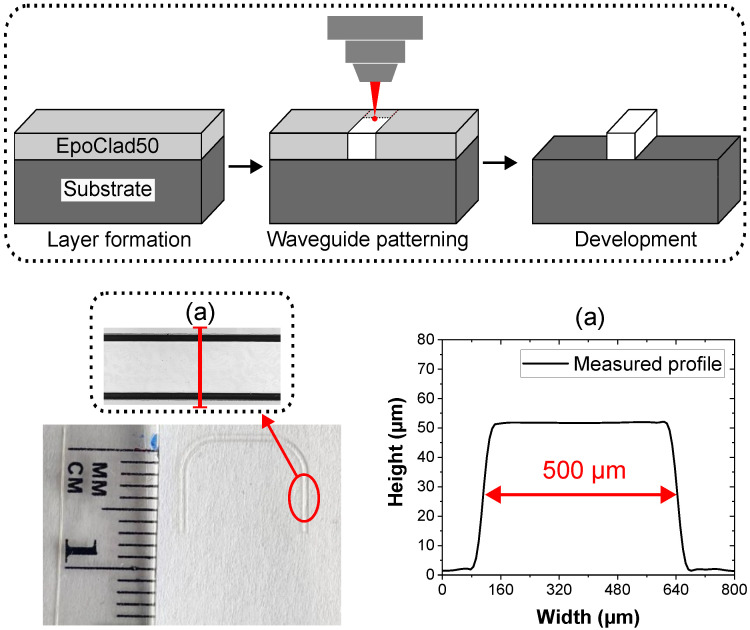
Above: Fabrication process of the PPW. Below: (left) Microscopic profile of the fabricated multimode PPW. (a) Cross-sectional profile of the PPW on a flexible substrate.

The proposed PPW-based tactile sensor foil utilizes a substrate of flexible cyclo-olefin co-polymer foil (mcs-foil-079, microfluidic ChipShop GmbH, Jena, Germany) with a thickness of 100 µm. The fabrication process of the PPW is schematically displayed within the dotted line area at the top of Figure 2.

The waveguide pattern was employed via laser direct photolithography technology—a maskless and rapid prototyping capable method [24]. At the beginning of the manufacturing process, the substrate foil was treated with oxygen plasma to create a hydrophilic surface, which optimizes adhesion prior to polymer application. Subsequently, the polymer resist EpoClad50 was deposited on the oxygen-activated surface using a spin-coater. The thickness of the resulting layer was adjusted by varying the speed of the spin-coating process. Once the 50 µm target was reached, and a series of heat treatments completed, the waveguide pattern was exposed using a photolithography machine (µPG 101, Heidelberg Instruments, Heidelberg, Germany). Afterwards, the unexposed polymers were removed with developer chemicals and the resulting waveguide structure was subjected to a final heat treatment. To determine the dimension of the fabricated structure, the waveguide was measured with a laser scanning microscope (VK-X250, Keyence Deutschland GmbH, Frankfurt am Main, Germany), the resulting waveguide profile is displayed in Figure 2a.

**Figure 3:**
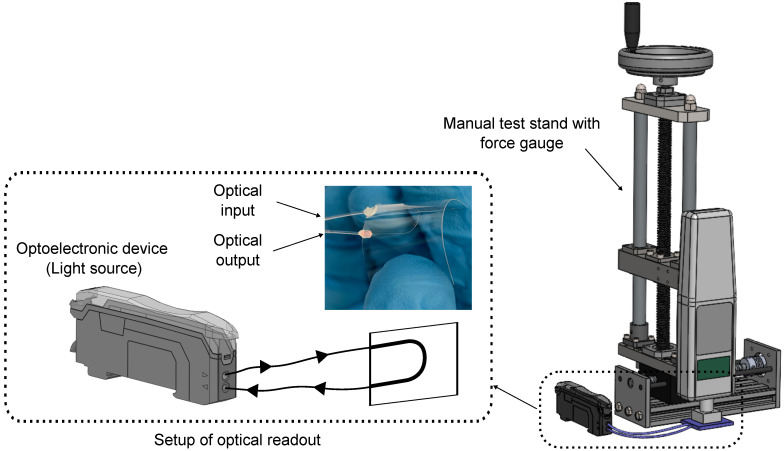
(**left**): Optical readout setup for the PPW sensor foil. The integrated photodiode within the optoelectronic device enables optical signal interrogation. (**right**): Experimental setup for characterizing PPW during tactile response. The setup includes a force gauge mounted on a manual test stand for referencing the contact force level (not up to scale).

Based on the sensing principle described in Section 2, optical signal measurements were conducted in transmission. In this configuration, POFs were used to transmit the optical signal between each component. Since the measurement principle relies on the quantification of optical intensity, it is essential to stabilize the emitted optical power from the light source to ensure reliable results. Accordingly, during the measurement process, the optical signal is generated by a digital optoelectronic device (Keyence fiber link FS-N40, Keyence Deutschland GmbH), which has a LED light source centered at a wavelength of 660 nm and an amplified photodiode [25]. The optical signal emitted by the light source is coupled into the PPW and the transmitted optical signal exiting the PPW is then directed to a photodetector unit for analysis, as shown in Figure 3 (left). A schematic of the optical signal measurement setup is presented inside the dotted line area of Figure 3.

To determine the PPW sensor’s performance in terms of light attenuation under applied force, the experimental setup displayed on the right side of Figure 3 was used. This setup includes a force gauge (FH20, Sauter GmbH, Breisgau, Germany) mounted on a manual test stand (TVL, Sauter GmbH), that enabled the controlled indentation of the waveguide. The applied force level is quantified by the force gauge.

## 4. Results and Discussion

### 4.1. Tactile Response Characterization

**Figure 4:**
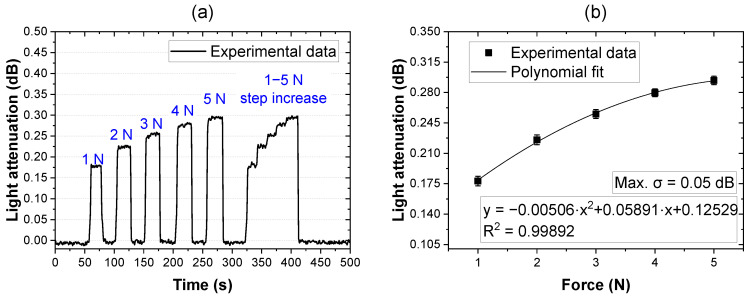
Light propagating behaviors and actuation due to bending loss. (**a**) Time-resolved readout of the sensor foil for contact forces between 1 and 5 N and stepwise increased impact with the same force levels. (**b**) Plotted light attenuation levels, the average values were fitted with a polynomial function.

The force sensing capability of the PPW sensor was evaluated using the experimental setup depicted in Figure 3. During the experiment, discrete static forces ranging from 1 to 5 N (representing the typical dynamic sensing range requirement of tactile sensors utilized in MIS applications [26]), were sequentially applied to the sensor, with each impacted force maintained for approximately 20 s. After each loading phase, the force was released to allow the light intensity to return to its initial level, confirming the reversibility of the sensor response. For each measurement cycle, the applied force was incremented in 1 N steps, ranging from 1 N to 5 N. Following the individual force application cycles, continuous force loading was conducted, in which the force was increased stepwise from 1 N to 5 N. The corresponding variations in optical intensity were monitored throughout this process to further evaluate the dynamic response of the PPW sensor due to incremental force loading. The sensor’s time-resolved optical response to a series of applied forces is presented in Figure 4a.

The time-resolved measurement data demonstrate a clear correlation between the magnitude of the applied force and the corresponding level of optical attenuation. Sequential application of forces from 1 N to 5 N produces distinct levels of optical attenuation, with each force eliciting an almost instantaneous sensor response, consistent for both individually and stepwise applied forces. As the magnitude of the applied force increases, a proportional rise in light attenuation is observed. Moreover, when a single force is increased stepwise from 1 to 5 N, the resulting attenuation values closely match those obtained from the separate, sequential force applications, demonstrating negligible hysteresis. To further evaluate the correlation between the applied force and sensor output, the monitored light attenuation values corresponding to different force levels are plotted in Figure 4b.

The overall sensor data can be fitted with a polynomial function, indicating a nonlinear sensor response. Notably, the sensitivity in attenuation per unit force is higher in the lower force domain (1–3 N). When the applied force exceeds 4 N, the sensitivity decreases, although the sensor continues to exhibit distinct responses to incremental force variations within the measured contact force range up to 5 N.

**Figure 5:**
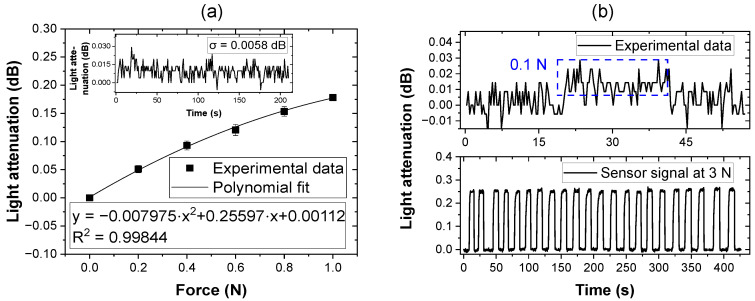
(**a**) Polynomial fit of the light attenuation response between 0 and 1 N in 0.2 N steps (inset: the noise of the readout without contact). (**b**) Above: Response of the sensor at contact force as low as 0.1 N; below: durability test of the sensor under repeated 3 N impact.

A detailed investigation into the sensor’s performance within the low-force area (0–1 N) was also conducted to further characterize the PPW sensor’s performance and detection capabilities [Figure 5a]. This time, contact forces were incrementally applied in 0.2 N steps up to a maximum of 1 N. The resulting attenuation response was again well illustrated by a polynomial fit, corroborating the nonlinearity observed in the broader force range. A theoretical minimal detectable force as low as 0.08 N was calculated from the standard deviation of the noise signal [0.0058 dB, displayed in the inset of Figure 5a].

As is apparent from the plotted data, a nonlinearity in the light attenuation response to externally applied force is observed. Overall, the response can be fitted by a polynomial function. This nonlinear behavior is similar to that found in previous studies [27], not only for optical intensity-based tactile sensors [15,18,20,28] but also for other transduction mechanisms such as electro-resistive tactile sensors [29], and magnetic tactile sensors [30], indicating a generalized tactile sensing characteristic across different sensor setups. In the case of the presented PPW, the nonlinearity can primarily be attributed to the unsymmetrical waveguide geometry. The ridge waveguide shape causes an anisotropic and inhomogeneous distribution of the effective refractive index inside the waveguide, which was analytically calculated by Huang [31]. Upon application of external forces, the experimental results depicted in Figure 4b and Figure 5a resemble the numerically calculated bending loss curves reported by Marcuse, who analyzed optical losses in asymmetric slab waveguides under bending deformations [32]. It can be assumed that a symmetric waveguide geometry, such as circular-shaped waveguides, would reduce the degree of nonlinearity in the bending response and promote a more uniform sensor output [33]. However, even with a symmetric waveguide design, external force loading will always induce local deformation of the waveguide core. This deformation disrupts the symmetry of the core under stress, regardless of the initial geometry, and leads to a complicated redistribution in the guided light modes. As a result, the optical intensity response to tactile input remains nonlinear, complicating the signal interpretation for tactile sensing. This phenomenon was simulated and experimentally demonstrated in the recent work by Yin and Ishigure, who used a circular, deformable waveguide core subjected to various external force loadings. Their results showed that as the waveguide deformed under increasing external force loads, the nonlinearity became more pronounced with larger core diameters, indicating a correlation between greater deformation and enhanced nonlinear behavior [34].

Despite the nonlinear optical response, our PPW remains suitable for practical tactile sensing applications, as the force response relationship is distinct and reproducible for each force magnitude. The influence of nonlinearity can be effectively addressed through calibration, allowing for compensation and normalization of the PPW’s output. Notably, in certain application scenarios, the integration of an additional mechanical actuator structure alongside the waveguide can enhance the sensitivity within a targeted force range, thereby improving the linearity of the tactile response over the desired dynamic sensing range [35,36,37].

Compared to other optical tactile sensors based on transmitted intensity measurements, which often experience near-total extinction of transmitted light (attenuations exceeding 10 dB) at applied forces as low as 5 N [15,34,38], the PPW shows a comparatively lower sensitivity. This reduced sensitivity can be mainly attributed to two principal factors. First, as discussed in Section 2, the bending loss increases nonlinearly with the waveguide core dimension (see Equation (1)). While the PPW fabricated with EpoClad50 achieved a core height of 50 μm in height, representing the upper limit of the employed photoresist, this is still smaller than that of although the dimension of conventional multimode POFs, which commonly exceed 500 μm diameter. Similarly, Amouzou et al. proposed in their most recent work a rectangular bend sensitive waveguide sensor based on PDMS material, a higher sensitivity is reported in relation to their increased waveguide core dimension (3 × 2 mm in width and height) [33]. Second, according to the simulation and the experiment carried out by Yin and Ishigure, the numerical aperture (NA) of the waveguide, determined by the refractive index contrast between the core and cladding materials, is inversely correlated to bending loss sensitivity. Specifically, a lower NA results in greater sensitivity to bending loss [34]. In our PPW, the ridge waveguide (RI = 1.58) is cladded by air (RI = 1), resulting in a high refractive index contrast and thus a high NA at the core–cladding interface. This high NA impedes the coupling of guided light from the waveguide core into the cladding, even under significant bending-induced curvature, thereby limiting the magnitude of bending loss that can be achieved through external force loading. Applying other materials around the waveguide consists of EpoClad may in this case enhance the sensitivity. However, this could also result in increased thickness of the overall sensor foil and reduce application flexibility, especially for non-planar surfaces.

In summary, it can be assumed that the contact force response of the PPW is primarily attributed to the bending of the flexible substrate and the PPW structure under external force loading. Given the high NA of the PPW configuration, optical coupling between the waveguide core (EpoClad) and the cladding (air on the upper side and substrate on the lower side) interface is minimal, and therefore not predominant to the sensor’s performance.

The minimum force detection capability of the sensor was also evaluated, revealing the contact force detection limit of 0.1 N, as shown in the upper side of Figure 5b. This detection limit is fundamentally constrained by the sensitivity of the receiving photodiode and the resolution of the analog-to-digital converter in the signal acquisition electronics. Using an analog-to-digital converter with greater bit depth could further enhance detection capabilities. In further quantifying our results, reproducibility and mechanical durability were evaluated through cyclic loading at a constant force of 3 N, as shown on the lower side of Figure 5b. The optical attenuation response to repeated force applications remained stable throughout the measurement period, exhibiting negligible hysteresis. Moreover, the optical intensity reliably returned to its baseline value after each unloading cycle, demonstrating the sensor’s robust performance under repeated tactile sensing applications.

### 4.2. Stiffness Sensing Characterization

To further explore the potential of the sensor foil for differentiating between various hardness levels, the sensor foil was attached to a 3D-printed indenter. To set defined stiffness levels for for the characterization of the performance of the sensor, a set of silicone dome-shaped samples was cast using 3D-printed molds with a mixture of two silicone materials (Ecoflex 00-20 and MoldMax 40, Smooth-On Inc., Macungie, PA, USA) were used in pre-determined ratios, resulting in hardness levels of 7, 14, 18, 22, 30 and 40 (Shore A), as characterized by a Shore A durometer (PCE-DD-A, PCE Instruments, Manchester, UK).

**Figure 6:**
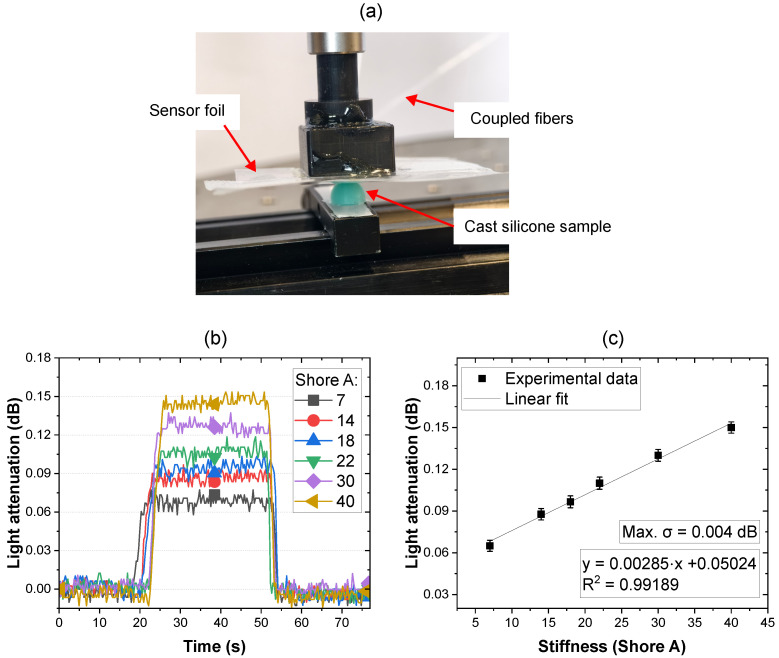
(**a**) Experimental setup for stiffness measurement. (**b**) Time-resolved readout of the sensor foil indenting silicone dome samples with varying Shore A stiffnesses. (**c**) Plotted levels of light attenuation with the average values of each level fitted with a linear function.

The sensor foil was indented onto different silicone samples of varying stiffnesses, as shown in Figure 6a. The indentation distance of each measurement was kept constant at 1 mm to ensure a reproducible light attenuation caused on the waveguide for each stiffness level. The monitored time-resolved readout induced by each stiffness is shown in Figure 6b.

The dependence of the sensor’s response on material stiffness is attributed to the variation in interaction force of both surfaces during indentation. As analyzed by Guzman et al., the resulted contact force correlates with material stiffness, with higher stiffness resulting in greater force upon indentation [39]. In the case of the sensor indentation upon different samples, soft materials dissipate compressive stress both vertically and laterally, resulting in a lower correlated contact force and reduced light attenuation. In contrast, harder materials undergo less deformation, generating a higher contact force and thus greater light attenuation.

The measurement results demonstrated a notable increase in light attenuation when the sensor foil indented the silicone dome sample. Distinct light attenuation levels were observed for each stiffness level. These are plotted in Figure 6c. Here, the light attenuation level corresponding to stiffness can be fitted with a linear function, and a sensitivity of 0.00285 dB/Shore A can be concluded. Compared to the tactile force characterization shown in Figure 4, which exhibited a non-linear response, a lower light attenuation response range was observed with maximial light attenuation of 0.15 dB being below the 0.3 dB seen in Figure 4, thereby contributing to the linearity. When applying tactile forces with a metal indenter, the indenter’s significantly greater stiffness relative to the waveguide and substrate materials results in light attenuation dominated by waveguide and substrate deformation. However, when indenting silicone samples of varying stiffness levels, both the sample and the PPW sensor deform upon contact, leading to a more complex dynamic interaction between two relatively soft surfaces [40]. The linear response observed may vary if a broader dynamic range is investigated.

### 4.3. Integrated Sensing Applications

#### 4.3.1. Stiffness Mapping

**Figure 7:**
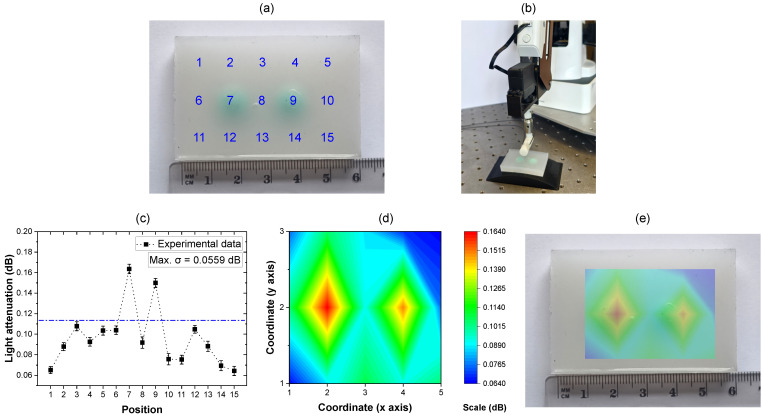
Stiffness mapping experiment set-up and results: (**a**) A Phantom sample fabricated to simulate tissue anomalies, the surface is labeled with sequentially numbers indicating the indenting positions. (**b**) PPW sensor foil integrated on a robot arm. (**c**) Monitored light attenuation in response to the indentation of the numbered positions on the phantom, the two stiffness levels can be separated by the blue dash-dot line. (**d**) Interpolated stiffness map with the x and y axes indicating the dimensions of the phantom and the z scale displaying the monitored stiffness levels. (**e**) The stiffness map overlaid onto the phantom.

In order to evaluate sensor performance, a silicone phantom sample was fabricated. Within it, two dome-shaped structures made of silicone material with Shore A-40 hardness (MoldMax 40, Smooth-On Inc.) were embedded into a layer of softer silicone of Shore 00-20 (Ecoflex 00-20, Smooth-On Inc.) to simulate tissue anomalies and mimic a biomedical application environment. Performance was evaluated by the sensor’s ability to identify and localize the two anomalies. The phantom’s dimensions were 6 × 4 cm (width by height), and its surface was labeled with 15 numbered positions, sequentially arranged to indicate targeted palpation sites [see Figure 7a].

The sensor foil was mounted on the lateral surface of a 3D-printed shaft module that had similar dimensions to a conventional endoscopic camera component with an outer diameter of 11 mm [41]. On the site where the sensor foil is positioned, a 1 mm gap was included into the design of the 3D-printed endoscopic camera module to accommodate the waveguide and enable sufficient space to allow microbending-based sensor actuation. The integrated sensor was horizontally mounted on a robotic arm (Dobot Magician, Shenzhen Dobot Corp., Ltd., Shenzhen, China) used to perform lateral indentation at each marked position on the phantom, applying a controlled indentation depth displacement of 1 mm at each numbered site. During the experiment, each labeled position on the phantom was held at the indented displacement for approximately 20 s, the resulted optical attenuation was monitored using the measurement setup described in Figure 2. The time-averaged light attenuation responses corresponding to each indentation are presented in Figure 7c.

Based on the measurement results, it was observed that each indentation applied to the phantom sample resulted in an increase in transmitted optical signal attenuation. When the sensor was robotically indented at positions corresponding to regions of softer material, the resulting optical attenuation remained below 0.111 dB. In contrast, indentations at positions 7 and 9, which correspond to locations of stiffer embedded structures, produced a obvious increase in optical attenuation relatively to other positions, with values of 0.167 dB and 0.153 dB, respectively. The maximal standard deviation of the monitored light attenuation during the 1 mm indented displacement was recorded as 0.0559 dB. These results demonstrate the sensor’s capability to discriminate between regions of varying material stiffness within a single phantom sample. For better visualization, the light attenuation data were plotted as a contour diagram. In this diagram, the x and y axes represent the spatial coordinates of the 15 indentation positions, arranged in a 3 × 5 matrix distributed along the xy-plane. The z axis corresponds to the intensity of monitored light attenuation. Higher attenuation values are seen as red while lower attenuation values are seen as green and blue. The resulting contour diagram is displayed in Figure 7d, providing clear spatial mapping of stiffness variations as detected by the sensor across the phantom sample.

The measurement results show that the observed light attenuation responses was greater than the characterized results presented in Figure 6. Notably, indentations at positions 7 and 9, corresponding to regions with a Shore A hardness of 40 as measured by durometer, produced maximum light attenuation of 0.167 dB, while the corresponding values in Figure 6 did not exceed 0.15 dB. Similarly, indentations in softer regions with hardness below Shore A-7 also produced higher attenuation levels compared to those reported in Figure 6b. This difference can principally be attributed to the integration of the sensor foil onto a non-planar surface in the robotic experimental setup. In the measurements shown in Figure 6b, the sensor was mounted planarly on a hollow indenter, limiting the contact area between the waveguide and the silicone samples during indentation. In contrast, within the robotic setup, the sensor foil was fixed to a curved surface with a diameter of 11 mm, resulting in the waveguide being pre-bent before indentation. This configuration also increased the interfacial contact area. In the previous setup, the dome-shaped silicone samples possessed 10 mm hemispherical bases, and the waveguide primarily interfaced with the tip of the hemisphere. During indentation, the soft phantom sample results in the lateral installed waveguide to partially immersed into it. The larger contact area is assumed to enhance the microbending-induced actuation, which in turn results in increased light attenuation. However, despite varying stiffness response levels on account of different sensor integration configurations, material stiffness can still be clearly discriminated by the monitored light attenuation response. The blue dash-dotted line in Figure 7c distinguishes between the two different stiffness levels, and the generated stiffness “heat map” displayed in Figure 7d also correctly identifies the higher stiffness locations. Finally, the achieved stiffness map is overlaid onto the phantom in Figure 7e, highlighting the potential in practical applications.

#### 4.3.2. Surface Texture Reconstruction

**Figure 8:**
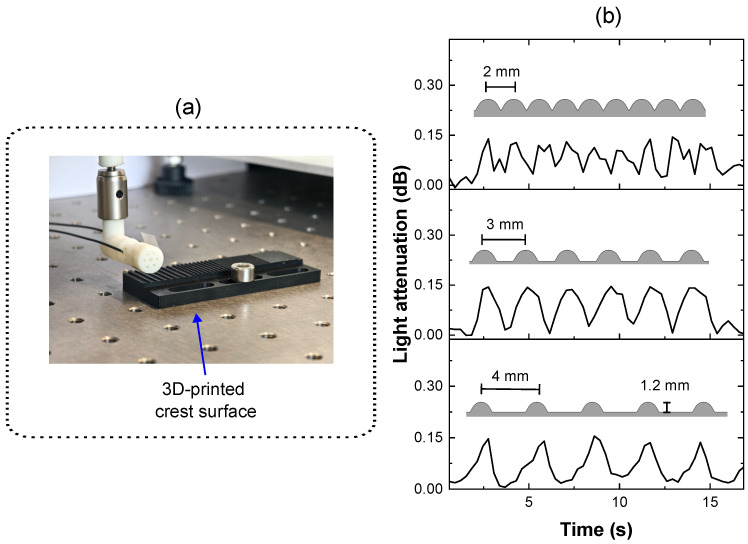
(**a**) Experimental setup for surface profile measurement. (**b**) Time-resolved readout of the sensor foil when passed over surfaces with varying roughness levels at the same velocity.

To assess the sensor’s ability to reconstruct surface texture profiles, a variety of rough surfaces, with 1.2 mm crest structures incorporated, were fabricated on a 3D-printed component. The different surface texture levels were defined by varying the distance between the crests by 2 mm, 3 mm and 4 mm, respectively. The tips of the crests were smoothed to prevent damage to the PPW. The measurement setup is shown in Figure 8a. During the measurement, the sensor integrated on the robotic arm was moved horizontally across the crest surface at a constant velocity. The time-resolved optical attenuation was continuously monitored and the reconstructed data are presented in Figure 8b.

As shown in Figure 8b, when the sensor foil was moved over a crest structure, the PPW experienced localized deformation upon contact, resulting in increased optical attenuation. As the sensor foil moved past the crest by the robot arm, the contact weakened and the deformation of the PPW reverted to its original shape, allowing the light attenuation to return to its baseline level. The time-resolved variations in light attenuation correspond to the spatial distances between crest structures, thus, the profile of the surface texture can be reconstructed from the time-resolved changes in optical attenuation.

From the measurement results, when the distance between crests was 3 and 4 mm, the time-resolved light attenuation closely resembled the crest surface texture profile. However, with 2 mm spacing, the light attenuation response was less clear. This limitation is likely attributable to the overall sensitivity of the employed optoelectronic device. It can be assumed that increasing the sampling frequency and sensitivity of the optical measurement system would in turn enhance the detection capability and sensitivity of the PPW sensor foil.

## 5. Discussion

The present study was conducted to evaluate the capability of a flexible sensor foil based on a PPW, designed for straightforward application without additional mechanical actuators or functional coatings, as a means of enhancing perceptual haptic stimulus assessment and compensating for the lack of haptic feedback encountered when utilizing surgical instruments during MIS. The demonstrated functionalities include the detection of varying contact forces, discrimination of material stiffnesses, and reconstruction of surface texture profiles. To summarize and evaluate the performance of the sensor relative to other research works, Table 1 presents a comparative analysis.

Compared to other optical intensity-based sensing technologies, the proposed sensor foil employs a simplified configuration, utilizing a commercially available optoelectronic device not specifically designed for photonic analysis. Although the PPW does not exhibit the highest absolute sensitivity among the compared approaches, the achieved performance is sufficient to demonstrate its potential for surgical palpation diagnostics. The nonlinear light attenuation response to tactile forces can be addressed through an appropriate calibration process. The thin, compact, and flexible characteristics of the PPW and substrate further facilitate versatile integration and positioning, enabling deployment on both frontal planar surfaces and lateral non-planar regions of surgical instruments.

Nevertheless, further enhancement of sensitivity remains a desirable outcome for future research. A detailed investigation of the mode field distribution within the waveguide can contribute to a better understanding of the power proportion inside the higher-order mode. For example, Yang et al. demonstrated improved surface plasmon resonance sensitivity by applying a mode-selective coupler [42]. Another potential method is to apply compressed air beneath the PPW sensor foil to generate a counterpressure on the opposite side of the substrate. This approach can increase the mechanical deformation of the PPW by external forces, and thereby increasing optical attenuation. Furthermore, the air pressure can be modulated, enabling an adjustable sensing dynamic range [25], this could be used for a fast, simplified calibration routine, if necessary.

The currently employed photolithographic fabrication technique constrains the waveguide geometry to a rectangular profile. As previously discussed, this may induce complex modal attenuation characteristics in response to tactile forces. To address this limitation, two-photon polymerization could serve as an alternative technique for the fabrication of symmetric waveguide geometry. Various studies have demonstrated the feasibility of optical waveguide sensors produced via the two-photon polymerization technique [43,44], which offers superior waveguide geometry resolution and additionally allows for more complex microstructural designs that may improve the reproducibility and stability of the sensor’s response [45]. The current experimental procedures for haptic stimulus assessment rely on controlled displacement, this may not always be feasible in surgical environments where precise control is often limited. In such scenarios, the accuracy of measurement could be negatively affected. It is anticipated that increasing the sensor foil’s sensitivity would allow for greater flexibility in measurement procedures. Moreover, the combination of PPW with other optical sensors, such as fiber Bragg gratings positioned at the frontal location of the MIS tool can also enhance overall sensor functionality and measurement accuracy by providing additional displacement information. For example, a Bragg grating-based sensor has been presented in our previous research for displacement measurement [12].

Future work will focus on adapting the sensor foil for integration into hand-held devices, thereby enabling reliable functionality under variable manual displacement conditions. Additionally, ambient temperature can affect the performance of PPW sensors. One effective mitigation approach involves integrating an optical splitter into the waveguide structure. This splitter divides the incoming light into two separate waveguides—one of which functions as a temperature reference sensor, exposed solely to the environmental conditions. The optical signals monitored from this reference path can then be used to compensate for temperature-induced variations in the primary sensor’s output. Also, the durability as well as the reliable repeatability of the sensor’s response requires more quantitative investigation in future studies. However, under the current design, the sensor is intended to be disposable following each surgical procedure.

For the application in MIS, the biocompatibility of the sensor material remains to be investigated. Although Hessler et al. validated the hemocompatibility of the EpoClad photoresist and cyclo-olefin copolymer substrate [46], the UV adhesives used for fixing the position of coupled POFs onto the waveguide may also be exposed to tissues during surgical operations and therefore, need to be evaluated in terms of biocompatibility for future commercial applications. However, this concern can be addressed by covering the entire sensor with a biocompatible barrier layer, which prevents direct contact between the sensor and human tissue during surgical procedures. Furthermore, the barrier layer can also provide hydrophobic and light absorbing (or reflecting) properties on the outer surface that is exposed to tissues, thereby shielding the PPW from the effects of environment moisture and ambient light, minimizing cross-sensitivity effects.

## 6. Conclusions

In this work, we have proposed a flexible sensor foil based on a planar polymer optical waveguide, and have demonstrated straightforward measurement capabilities for contact force, material stiffness, and surface texture reconstruction. For contact force sensing, the sensor achieved a measurable resolution of 0.1 N within the range of 0–5 N. In terms of stiffness sensing, the sensor exhibited a response of 0.00285 dB/Shore A. Furthermore, integration of the sensor foil onto a robotic arm enabled stiffness mapping, localization of regions with varying stiffnesses of a phantom sample, and surface texture reconstruction. These results demonstrate the sensor foil’s potential to compensate for the lack of haptic feedback experienced by surgeons during MIS.

## Figures and Tables

**Table 1:** Performance comparison of the PPW sensor foil with other representative works based on evaluation of optical signal intensity.

Sensor Type	Range (Resolution)	Size (Width × Thickness)	Application	Comment / Key Limitation	Reference
PPW	0–5 N (80 mN)	10 × 0.15 mm	Contact force, material stiffness, surface texture	Waveguide on flexible substrate, straightforward measurement without additional coating or actuator. / Limited sensitivity.	This work
Rectangular POF	0–10 N (N.A.)	3 × 2 mm	Contact force, pressure	POF with porous cladding. / Anisotropic response.	Amouzou (2025) [33]
POF knot	0–10 N (N.A.)	5× 1 mm	Tri-axial force, slip and friction	POF actuated with knot structure. / PDMS encapsulation required.	Pan (2023) [18]
Coated POF	0–6 N (2.5 mN)	15 × 2 mm	Finger press, respiration, blood pulse, contact force, material stiffness, surface texture	Gold nanoparticles-coated POF actuated by a PDMS block sandwiched between two substrate layers. / Additional coating and actuator required.	Guo (2023) [15]
Micro/nanofiber	0–2.1 Pa (N.A.)	Not specified, fiber diameter 900 nm	Contact force, pressure, bending, acoustic vibration	Glass micro/nanofibers embedded in PDMS film. / Risk of cross-sensitivity.	Zhang (2020) [36]
Hydrogel optical fibers	0–0.175 N (N.A.)	1 × 0.03 mm	Pressure, contact force, ultrasonic and audible wave	PDMS coated hydrogel fiber embedded in PDMS layer, designed for underwater applications. / Small sensing dynamic range.	Wang (2020) [35]
Planar waveguide with actuator slide	0–3 N (N.A.)	Not specified, thickness < 0.15 mm	Dynamic input force (multi-positions)	Waveguide core separated by spacer, actuated by additional touch layer. / Complicated fabrication.	Yun (2014) [20]

## Data Availability

Data underlying the results presented in this paper are not publicly available but may be obtained from the authors upon reasonable request.

## Abbreviations

The following abbreviations are used in this manuscript:

NA	Numerical aperture
TIR	Total internal reflection
PPW	Planar polymer (optical) waveguide
POF	Polymer optical fiber
